# Immunotherapy benefits for large brain metastases in non-small cell lung cancer

**DOI:** 10.1093/oncolo/oyae314

**Published:** 2024-11-15

**Authors:** Narayanan Sadagopan, Edina Komlodi-Pasztor, Irina Veytsman

**Affiliations:** MedStar Georgetown Cancer Institute, Department of Hematology and Oncology, Washington, DC 20010, United States; MedStar Georgetown Cancer Institute, Department of Hematology and Oncology, Washington, DC 20010, United States; MedStar Georgetown Cancer Institute, Department of Hematology and Oncology, Washington, DC 20010, United States

**Keywords:** brain metastases, immunotherapy, non-small cell lung cancer, real-world data

## Abstract

**Introduction:**

Non-small cell lung cancer (NSCLC) patients with large brain metastases (BrM) defined as >2 cm in diameter historically face grim prognoses. With immunotherapy emerging as a promising avenue for BrM management and being commonly used in NSCLC, its application in addressing large BrM remains underexplored.

**Methods:**

This retrospective study conducted across the MedStar Georgetown Cancer Network aimed to assess the efficacy of immunotherapy in non-biomarker driven NSCLC patients with large BrM following initial treatment.

**Results:**

Thirty-six patients were included, all of whom underwent neurosurgery and/or radiation before commencing immunotherapy. The median intracranial progression-free survival (PFS) was 9.2 months and the median overall survival (OS) reached 31 months. Utilizing multivariable Cox penalized regression, the intracranial PFS hazard ratio (HR) was 0.07 (95% confidence interval (CI), 0.02-0.26) for patients who received at least 90 days of immunotherapy compared to those who did not. Each additional 30 days of immunotherapy was associated with an OS HR 0.77 (95% CI, 0.67-0.90).

**Conclusion:**

This real-world data highlights the potential of immunotherapy in large BrM NSCLC patients, a population often excluded from clinical trials. This study contributes insights that can inform future treatment approaches, emphasizing the need for further exploration of immunotherapy’s role in enhancing outcomes for this challenging patient population.

## Introduction

For metastatic non–small cell lung cancer (NSCLC), about 25%-30% of the patients will have a BrM at the time of initial diagnosis and have a 13% annual incidence of developing a BrM.^1^ The development of BrM has significant implications for quality of life as well as clinical trial eligibility and delays in systemic treatment. Historically, NSCLC patients with BrM had a poor median overall survival (OS) of 12.2 in a retrospective study using 2010-2015 data.^2^ However, a more recent single-center retrospective study with 126 NSCLC BrM patients had a similar median OS of 18 months compared to 18.7 months in NSCLC patients with extracranial metastases.^3^

Large BrM are defined as at least 2 cm in diameter based on toxicities associated with radiation.^4,5^ Prospective data on NSCLC patients with large brain metastases (BrM) is limited, as trials often exclude symptomatic or untreated, non-biomarker-driven BrM. The guideline consensus is to treat them with neurosurgery and/or radiation.^6^ One prospective trial of pembrolizumab in 18 NSCLC patients with asymptomatic BrM < 2 cm had a 33% objective response rate (ORR).^7^ The Atezo-Brain study included untreated asymptomatic NSCLC BrM patients. Intracranial progression-free survival (PFS) was 6.9 months and OS 11.8 months. It is unclear how many of the 40 patients had a large BrM as the median of the sum of all target brain lesions was 13 mm (range 10-42 mm).^8^ Immunotherapy has shown durable responses in the brain with 1 example being nivolumab plus ipilimumab having 5-year intracranial PFS of 16% compared to 6% for chemotherapy and less development of new BrM (4% vs 20%) in patients with previously treated BrM despite patients only receiving up to 2 years of immunotherapy.^9^ Given the documented efficacy of immunotherapy in smaller BrM, there is a potential for its application as a maintenance therapy in patients with large BrM.

## Methods

We retrospectively reviewed electronic medical records (EMR) across 3 MedStar Georgetown Cancer Institute sites for patients aged 18 and older with non-biomarker driven NSCLC diagnosed between January 01, 2017 and December 31, 2022 who developed a BrM of at least 2 cm in 1 dimension and started immunotherapy after diagnosis of the large BrM. Exclusion criteria were multiple primary cancers in the past 5 years, EGFR mutation, or ALK mutation. Extracranial and intracranial responses were assessed using RECIST 1.1 criteria from original imaging studies, with MRI used for brain imaging. Intracranial progression-free survival (PFS) was defined as the time from initial diagnosis of the BrM on imaging to intracranial progression or death. OS was defined as time from initial diagnosis of the BrM on imaging to death. Due to variable documentation in the EMR, treatment toxicity and steroid use were not analyzed. Due to the number of qualifying patients (36) and large number of predictors (12), power analysis was not able to be conducted with instead Cox penalized regression being used to assess intracranial PFS and OS. The study was approved by the Georgetown-MedStar institutional review board.

## Results

### Baseline characteristics

Of 190 patients who had NSCLC with BrM, 123 were excluded for not having a BrM of at least 2 cm and 31 did not meet other criteria leaving 36 patients with non-biomarker-driven NSCLC who had a BrM of at least 2 cm and started immunotherapy after the large BrM diagnosis. The median duration of follow-up was 17.6 months (95% CI, 7.13-22.22). The study population was slightly more female (*n* = 21, 58.3%), 38.9% African-American (*n* = 14), mostly received pembrolizumab for immunotherapy (*n* = 33, 91.7%), received an average of 288.1 days of immunotherapy (median 178 days), and had an average age of 64.5 years (Table 1). Both PD-L1 low < 1% and PD-L1 high > 49% groups had 15 patients. Thirty-four patients received stereotactic radiosurgery (SRS) while 2 patients underwent whole-brain radiation therapy (WBRT). Before starting immunotherapy, 12 patients received only radiation while 24 underwent neurosurgery followed by radiation.

**Table 1.** Potential predictors of PFS and OS. Data are mean ± 1 SD; *n* (%) for categorical predictors.

Predictor variable	Summary statistic
Ethnicity	
Caucasian	19 (52.8)
African-American	14 (38.9)
Asian	2 (5.6)
Other	1 (2.8)
Gender	
Male	15 (41.7)
Female	21 (58.3)
Histology	
Adenocarcinoma	28 (77.8)
Squamous cell	3 (8.3)
Carcinoma	3 (8.3)
Large cell	2 (5.6)
Largest brain lesion (cm)	
2.0-2.9	20 (55.6)
3.0-3.9	11 (30.6)
≥4.0	5 (13.9)
Number of brain lesions	
1	18 (50.0)
2-3	11 (30.6)
≥4	7 (19.4)
BrM treatment	
Radiation	12 (33.3)
Surgery plus radiation	24 (67.7)
Immunotherapy agent	
Pembrolizumab	33 (91.7)
Atezolizumab	2 (5.6)
Durvalumab	1 (2.8)
Concurrent chemotherapy with immunotherapy	
Yes	31 (86.1)
No	5 (13.9)
PD-L1	
<1%	15 (41.7)
1-49%	6 (16.7)
>49%	15 (41.7)
Age at diagnosis of BrM	64.5 ± 8.9
Days to immunotherapy start from BrM diagnosis	66.3 ± 37.3
Duration of immunotherapy (days)	288.1 ± 289.6

Total *n* for the denominator of percentages = 36.

### Outcomes

**Figure 1.**
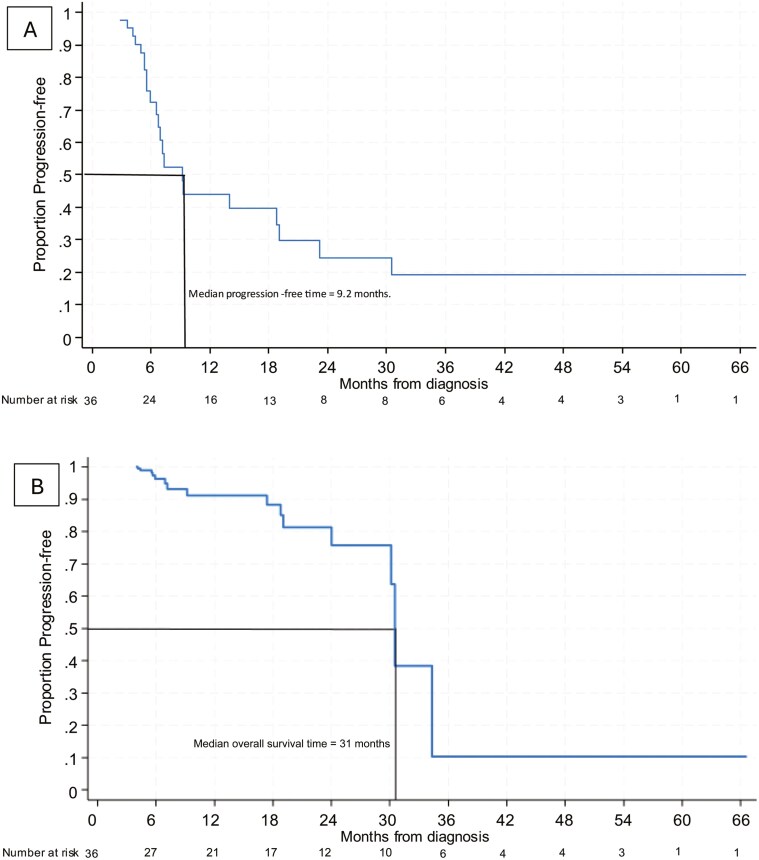
(A) Kaplan–Meier curve of intracranial PFS. (B) Kaplan–Meier curve of OS.

The median intracranial PFS was 9.2 months and the median OS was 31 months (Figure 1). Of the patients who had evidence of intracranial progression prior to death or last contact, 1 patient had progression of a target lesion while 11 patients developed a new brain lesion. The intracranial ORR at 6 months was 63.9% with 36.1% of the patients having a complete response (CR) (Supplementary Table S1). The extracranial ORR at 6 months was 52.8% (Supplementary Table S1). The 5 patients with oligometastatic intracranial disease had an extracranial CR following the resection of their primary lung tumor. Of the 12 predictors in Table 1, the 3 most associated with intracranial PFS were a number of brain lesions, duration of immunotherapy, and days to immunotherapy start (Supplementary Table S2). Of note, patients who received 90 or more days of immunotherapy had an intracranial PFS hazard ratio (HR) of 0.07 (95% CI, 0.02-0.26) compared to patients who received less than 90 days of immunotherapy (Supplementary Figure S1). The 3 predictors most associated with OS were gender, number of brain lesions, and duration of immunotherapy (Supplementary Table S3). Each additional 30 days of immunotherapy corresponded to an OS HR of 0.77 (95% CI, 0.67-0.90) (Supplementary Figure S2).

## Discussion

Our retrospective analysis yielded compelling results regarding outcomes of non-biomarker-driven NSCLC patients with large BrM, showcasing a real-world median intracranial PFS of 9.2 months and median OS of 31 months. While there are limitations in comparing studies, the median OS was quite different compared to 18 months seen in other retrospective studies.^3,10^ The extracranial ORR of 52.8% for our patients was similar to the 47.6% seen in KEYNOTE-189 in metastatic NSCLC suggesting that our patient cohort may not inherently harbor less aggressive disease.^11^ Although the sample size is small, our data challenges the notion that large BrM patients have uniformly poor outcomes.

Crucially, the duration of immunotherapy emerged as a key determinant of both OS (HR 0.77) and intracranial PFS (HR 0.07). However, we acknowledge the necessity for patients to survive longer to receive extended immunotherapy, potentially confounding the observed benefits. Another limitation is that all the patients received upfront treatment making it challenging to differentiate the benefits of immunotherapy from those of the upfront local treatment.

Interestingly, PD-L1 status and upfront BrM treatment were not among the top predictors associated with OS or intracranial PFS. The relatively limited role of the PD-L1 status aligns with the observed efficacy of pembrolizumab across all PD-L1 categories in metastatic NSCLC, as demonstrated in KETNOTE-189.^11^ Examining the outcomes of the 24 patients who underwent neurosurgery plus radiation, our study did not identify major concerns with this approach, although adverse events were not systematically analyzed. It is noteworthy that neurosurgery alters the treatment timeline for chemotherapy or immunotherapy. Prospective studies focusing on NSCLC patients with large BrM are essential to compare various upfront BrM treatment approaches. One such study tested nivolumab and ipilimumab with concurrent SRS in 13 NSCLC BrM patients showing an estimated 4-month intracranial PFS of 70.7%, but only 2 of the patients had large BrM with a maximum size of 2.2 cm.^12^ With many large BrM requiring steroids, it is an important consideration as steroids decrease the efficacy of immunotherapy.^13^ Variable EMR documentation prevented steroid use analysis in our study.

In conclusion, our study provides valuable insights into the favorable outcomes achievable for NSCLC patients with large BrM through an extended duration of immunotherapy. However, the challenge lies in conducting trials to isolate the specific impact of immunotherapy in this population, given the imperative for rapid relief through a neurosurgery intervention or radiation. Future directions should focus on clinical trials investigating the optimal timing of immunotherapy relative to upfront BrM treatment, with a specific emphasis on understanding the role of steroid dosing in this context.

## Supplementary material

Supplementary material is available at *The Oncologist* online.

oyae314_suppl_Supplementary_Table_S1

oyae314_suppl_Supplementary_Table_S2

oyae314_suppl_Supplementary_Figure_S1

oyae314_suppl_Supplementary_Table_S3

oyae314_suppl_Supplementary_Figure_S2

## Data Availability

Data cannot be shared for ethical/privacy reasons.
