# Supplementary material for: Immunotherapy benefits for large brain metastases in non-small cell lung cancer
Source: Oncologist. 2024 Nov 15;30(8):oyae314. doi: 10.1093/oncolo/oyae314 (PMC12395241; doi:10.1093/oncolo/oyae314)
Supplement: oyae314_suppl_Supplementary_Table_S1 [file oyae314_suppl_supplementary_table_s1.docx]

| **Response Rate** | **Partial Response** | **Complete Response** | **Objective response rate** |
| --- | --- | --- | --- |
| **Intracranial at 6 months** | 10 (27.7%) | 13 (36.1%) | 23 (63.9%) |
| **Extracranial at 6 months** | 13 (36.1%) | 6 (16.7%) | 19 (52.8%) |

Supplemental Table S1: Response rates 6 months from large BrM diagnosis
