# Supplementary material for: Immunotherapy benefits for large brain metastases in non-small cell lung cancer
Source: Oncologist. 2024 Nov 15;30(8):oyae314. doi: 10.1093/oncolo/oyae314 (PMC12395241; doi:10.1093/oncolo/oyae314)
Supplement: oyae314_suppl_Supplementary_Table_S2 [file oyae314_suppl_supplementary_table_s2.docx]

| **Predictor** | **Hazard Ratio** | **95% Confidence Interval** |
| --- | --- | --- |
| **Number of brain lesions** |  |  |
| 1 | 1.00 | --------------- |
| 2-3 | 2.30 | 0.79 – 6.73 |
| ≥ 4 | 2.66 | 0.86 – 8.29 |
| **Duration of immunotherapy** |  |  |
| < 90 days | 1.00 | --------------- |
| ≥ 90 days | 0.07 | 0.02 – 0.26 |
| **Days to start of immunotherapy** | 0.47 | 0.26 – 0.84 |

Supplemental Table S2: Hazard ratios for intracranial PFS for top three predictors. Hazard ratio 1 indicates reference category. Days to immunotherapy start in 30-day increments.
