# Supplementary material for: Immunotherapy benefits for large brain metastases in non-small cell lung cancer
Source: Oncologist. 2024 Nov 15;30(8):oyae314. doi: 10.1093/oncolo/oyae314 (PMC12395241; doi:10.1093/oncolo/oyae314)
Supplement: oyae314_suppl_Supplementary_Table_S3 [file oyae314_suppl_supplementary_table_s3.docx]

| **Predictor** | **Hazard Ratio** | **95% CI** |
| --- | --- | --- |
| **Female** | 2.38 | 0.50 – 11.26 |
| **Number of brain lesions** |  |  |
| 1 | 1.00 | --------------- |
| 2-3 | 1.52 | 0.36 – 6.40 |
| ≥ 4 | 6.92 | 1.50 – 32.00 |
| **Duration of immunotherapy** | 0.77 | 0.67 – 0.90 |

Supplemental Table S3: Hazard ratios for OS for top three predictors. Hazard ratio 1 indicates reference category. Duration of immunotherapy in 30-day increments.
