# Supplementary material for: Immunotherapy benefits for large brain metastases in non-small cell lung cancer
Source: Oncologist. 2024 Nov 15;30(8):oyae314. doi: 10.1093/oncolo/oyae314 (PMC12395241; doi:10.1093/oncolo/oyae314)
Supplement: oyae314_suppl_Supplementary_Figure_S2 [file oyae314_suppl_supplementary_figure_s2.docx]

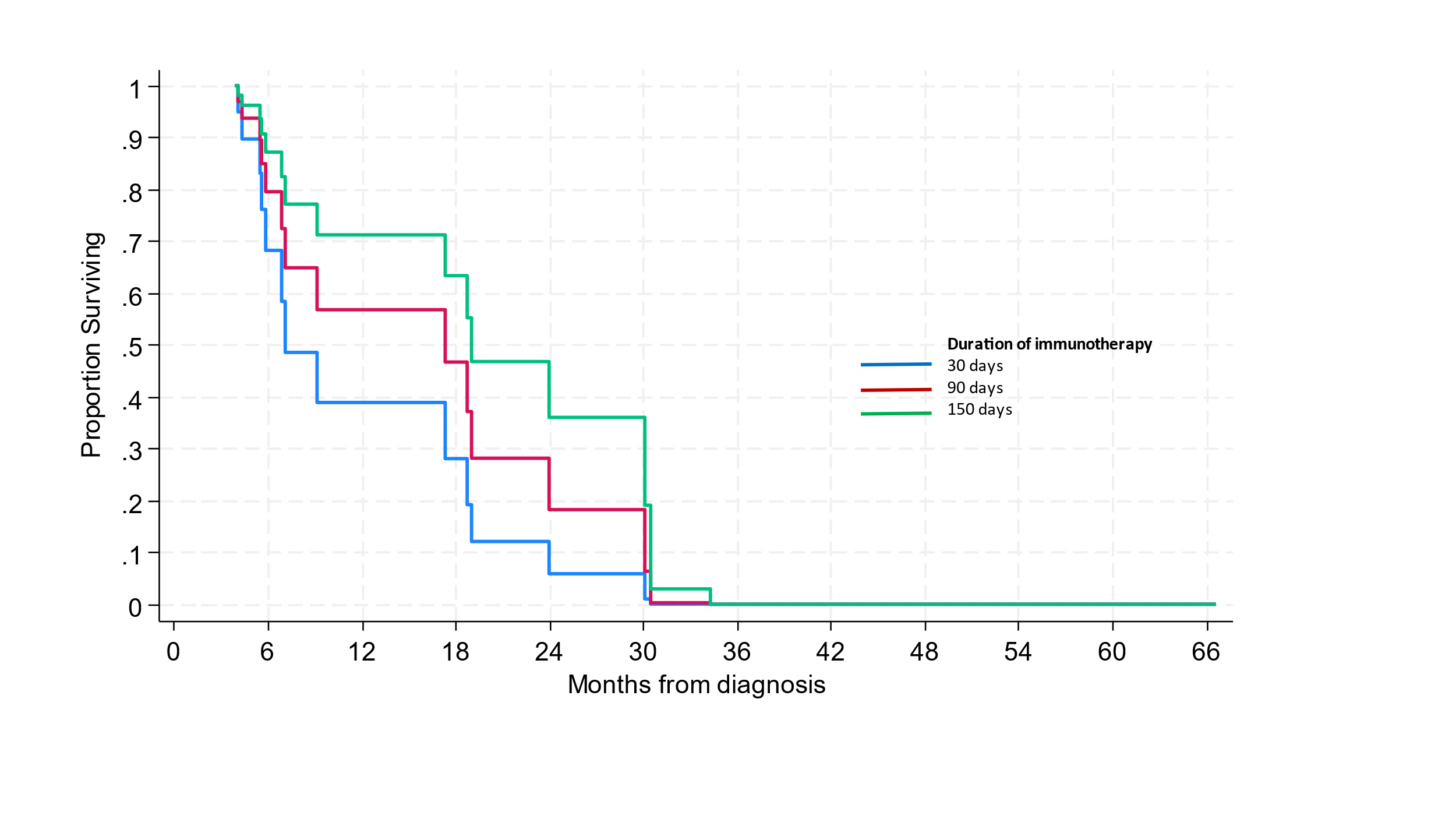


Supplemental Figure S2: OS Cox proportional hazards regression by duration of immunotherapy adjusted for sex and number of lesions
